# Geographic variation in advertisement calls of a Microhylid frog – testing the role of drift and ecology

**DOI:** 10.1002/ece3.2116

**Published:** 2016-04-12

**Authors:** Ko‐Huan Lee, Pei‐Jen L. Shaner, Yen‐Po Lin, Si‐Min Lin

**Affiliations:** ^1^ Department of Life ScienceNational Taiwan Normal UniversityTaipeiTaiwan; ^2^ Division of ZoologyTaiwan Endemic Species Research InstituteNantouTaiwan

**Keywords:** acoustic signal, advertisement call, *Microhyla*, prezygotic isolation, reproductive character displacement

## Abstract

Acoustic signals for mating are important traits that could drive population differentiation and speciation. Ecology may play a role in acoustic divergence through direct selection (e.g., local adaptation to abiotic environment), constraint of correlated traits (e.g., acoustic traits linked to another trait under selection), and/or interspecific competition (e.g., character displacement). However, genetic drift alone can also drive acoustic divergence. It is not always easy to differentiate the role of ecology versus drift in acoustic divergence. In this study, we tested the role of ecology and drift in shaping geographic variation in the advertisement calls of *Microhyla fissipes*. We examined three predictions based on ecological processes: (1) the correlation between temperature and call properties across *M. fissipes* populations; (2) the correlation between call properties and body size across *M. fissipes* populations; and (3) reproductive character displacement (RCD) in call properties between *M. fissipes* populations that are sympatric with and allopatric to a congener *M. heymonsi*. To test genetic drift, we examined correlations among call divergence, geographic distance, and genetic distance across *M. fissipes* populations. We recorded the advertisement calls from 11 populations of *M. fissipes* in Taiwan, five of which are sympatrically distributed with *M. heymonsi*. We found geographic variation in both temporal and spectral properties of the advertisement calls of *M. fissipes*. However, the call properties were not correlated with local temperature or the callers' body size. Furthermore, we did not detect RCD. By contrast, call divergence, geographic distance, and genetic distance between *M. fissipes* populations were all positively correlated. The comparisons between phenotypic *Q*_st_ (*P*_st_) and *F*_st_ values did not show significant differences, suggesting a role of drift. We concluded that genetic drift, rather than ecological processes, is the more likely driver for the geographic variation in the advertisement calls of *M. fissipes*.

## Introduction

Acoustic signals are critical to species recognition and mate competition (Claridge and de Vrijer 1994; Ritchie 2007). Rapid changes in acoustic signals due to genetic recombination (Vargas‐Salinas and Amézquita 2013), cultural learning (Lachlan and Servedio 2004), and/or local adaptation (Morton 1975; Ziegler et al. 2011) have been documented, with broad implications in population differentiation and contemporary speciation. The divergence in acoustic signals facilitates and reinforces speciation processes (Seddon et al. 2013; Wilkins et al. 2013; Mendelson et al. 2014); on the other hand, it may exacerbate negative impacts of habitat fragmentation by reducing gene flows among isolated populations (Hitchings and Beebee 1997; Cushman 2006). Therefore, it is important to understand the drivers of divergent evolution in acoustic signals, particularly those used for reproduction such as advertisement calls.

Advertisement calls, as for any trait, diverge as a result of deterministic (selection) and/or random (drift) processes. Selection can act upon call properties directly or indirectly through correlated traits. Many ecological factors are known to influence call transmission (e.g., temperature, habitat structure, background noise, competition in call space), and therefore, local adaptation can directly drive call divergence (Wilkins et al. 2013). Furthermore, morphological traits such as body size are known to constrain call properties (Ophir et al. 2010). Consequently, selection for certain morphology can have an indirect effect on call divergence. Selection processes that involve only one target species can be probed by examining the correlations between ecological conditions (e.g., temperature) and call properties (or their correlated traits such as body size) of the species across their geographic populations. However, selection processes that involve interactions between closely related species are more difficult to detect. For example, frogs that participate in mixed‐species choruses may be under selection to avoid interspecific competition for signal space (Chek et al. 2003; Amézquita et al. 2006) and heterospecific mating (Gerhardt 1994; Moriarty and Lemmon 2010). This could lead to reproductive character displacement (RCD; Brown and Wilson 1956; Pfennig and Pfennig 2012), where advertisement calls should become more differentiated in sympatric than in allopatric zones for pairs of closely related species. Such reproductive character displacement has been reported in tree frogs: The dominant frequency of American green tree frogs (*Hyla cinerea*) becomes higher when sympatrically distributed with the closely related barking tree frogs (*H. gratiosa*) (Höbel and Gerhardt 2003). Therefore, it is important to examine the presence of RCD in order to determine the role of biotic interaction as an ecological driver for call divergence.

Despite the many possible ecological drivers, call divergence can still be a drift process (Mayr 1963; Irwin et al. 2008; Amézquita et al. 2009; Campbell et al. 2010). However, given that selection is widely believed to be the main force underlying species diversity, the role of genetic drift in call divergence is often ignored compared to the role of ecology (Amézquita et al. 2009). In fact, genetic drift, working in concert with selection, has been shown capable of driving speciation (Uyeda et al. 2009). Therefore, the role of drift in the divergence of traits that are critical to speciation process warrants more studies. Genetic drift is a random process generated through “sampling error,” leading to stochastic fluctuations in allele frequencies. Isolation by distance is a phenomenon caused by genetic drift when the geographic barrier between populations is too far for dispersal, leading to a positive correlation between genetic and geographic distances (Wright 1943). Therefore, positive correlations between trait divergence, genetic distance, and geographic distance indicate that the trait is likely under the influence of genetic drift. To evaluate the role of drift in call divergence, one must look at the correlations between call divergence, genetic distance, and geographic distance, as well as the evidence for potential ecological divers, such as temperature, body size, and RCD.

**Figure 1:**
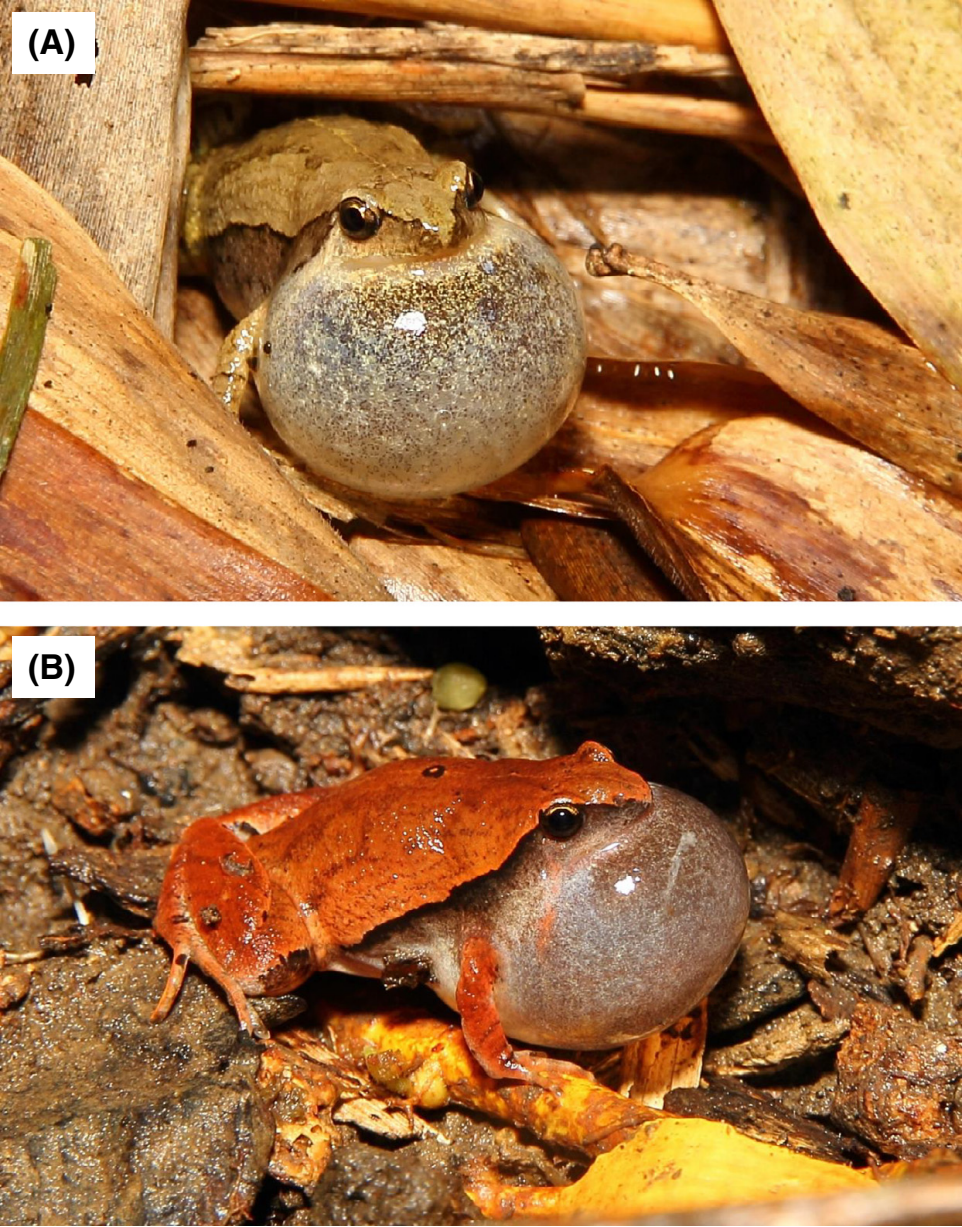
(A) *Microhyla fissipes* Boulenger, 1884; and (B) *Microhyla heymonsi* Vogt, 1911. Photographed by Jia‐Ming Tsao.

Amphibians are known for their low vagility and are easily separated by geographic barriers (Monsen and Blouin 2004; Funk et al. 2005; Guarnizo et al. 2009). Advertisement calls of frogs, playing a critical role in sexual selection and species recognition, are one of the best models to study divergent evolution in acoustic signals. In this study, we tested the role of ecology and drift in shaping geographic variation in the advertisement calls of a microhylid frog. *Microhyla fissipes* Boulenger, 1884 (Fig. 1A), which distributes widely throughout the low lands of Taiwan. Geographically, they overlap with a closely related congener *M. heymonsi* Vogt, 1911 (Fig. 1B) in their southern range. These two species produce advertisement calls which are indistinguishable to humans (Kuramoto 1987). Furthermore, both species form explosive‐breeding assemblages in mixed‐species chorus, making them good candidates for RCD.

The topography of Taiwan is extremely steep with many rivers flowing from high mountains to the coast, creating a highly fragmented landscape, which likely contributed to the high levels of differentiation in many terrestrial species (Lin et al. 2012; Tseng et al. 2015; You et al. 2015). Taiwan is a relatively small island in the subtropical region, with latitudes and longitudes spanning less than 3 degrees (22°–25°N, 120°–122°E). As a result, the climate gradient is generally shallow for low land species (e.g., the differences in monthly mean temperature range between 1°C and 4°C between the northern city Taipei and the southern city Kaohsiung; the Central Weather Bureau in Taiwan). These characteristics make the island an ideal system to test the role of genetic drift in shaping geographic variation in acoustic signals.

We first examined geographic variation in the advertisement calls and body size (a trait likely constrains call properties and be subject to direct selection) of *M. fissipes*. To test the role of ecology, we examined: (1) the correlations between long‐term average temperature and call properties across *M. fissipes* populations; (2) the correlations between call properties and body size across *M. fissipes* populations; and (3) RCD between *M. fissipes* populations that are sympatric with and allopatric to *M. heymonsi*. To test the role of drift, we examined the correlations between geographic distance, genetic distance, and call divergence across *M. fissipes* populations.

## Materials and Methods

### Sampling design

**Figure 2:**
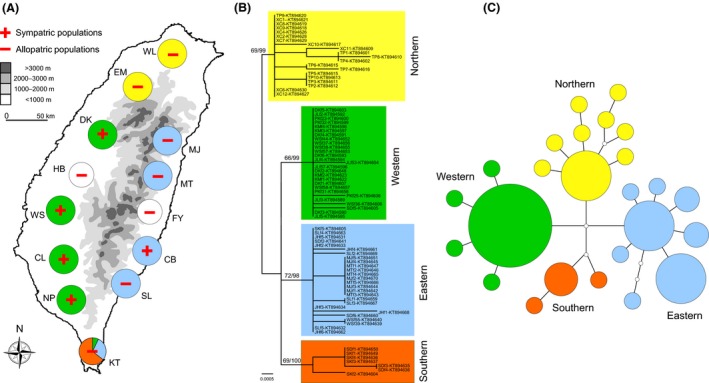
Sample localities and phylogenetic structure of *Microhyla fissipes*. (A) Distribution of the 11 sample localities of *M. fissipes*, including five sympatric populations (DK, WS, CL, NP, and CB), and six allopatric populations (WL, EM, KT, SL, MT, and MJ). Two additional sample localities (HB, FY) where only *M. heymonsi* occurs were also included in this study to provide a more complete quantitative description of the call properties of these two *Microhyla* species (Appendix S1). Different colors correspond to the four clades of *M. fissipes* haplotypes of mitochondrial *COI* sequences, which was recovered by maximum likelihood tree (B) and minimum spanning network (C).

A total of 146 *M. fissipes* samples were collected from 11 geographic populations (five sympatric with and six allopatric to *M. heymonsi*; Fig. 2A & Appendix S1) in Taiwan between March and August of 2014. We captured adult males at each site after the advertisement call recording (see Advertisement call recording and analyses). Upon capture, each individual was placed into a plastic ziplock bag for morphological measurement. All individuals were weighed to the nearest 0.01 g, and their snout‐vent lengths (SVLs) were measured to the nearest 0.01 mm using a digital caliper (Mitutoyo, Kanagawa, Japan). For genetic analysis, we clipped one of the frogs' hind leg toes and preserved it in 95% ethanol. We also recorded ambient temperature using a thermos‐hygrometer (Lutron, Taipei, Taiwan) at the same time of the call recording. We released all frogs to where they were captured at immediately after the sampling. We recorded the latitude, longitude, and elevation of each locality using a GPS receiver (Oregon 550t, GARMIN Ltd, Taipei, Taiwan). We extracted long‐term (1960–2009) monthly average temperature data between March and August from the Taiwan Climate Change Projection and Information Platform (http://tccip.ncdr.nat.gov.tw/v2/index_en.aspx) using the localities' geographic coordinates (Appendix S1).

### Advertisement call recording and analyses

Advertisement calls were recorded at a rate of 44.1 kHz, 16‐bit resolution using a digital recorder (Sony PCM‐M10, Tokyo, Japan) with a shotgun microphone (Sony ECM‐CG50), which was placed as close to the caller as possible. We recorded at least 20 consecutive calls from each individual and used Raven pro v1.4 (Cornell Lab of Ornithology, Ithaca, NY) to characterize the acoustic traits.

*Microhyla fissipes* typically produce a series of calls consecutively, with each call composed of numerous pulses (Appendix S2). For the temporal properties, we measured call duration, call interval, call rate, call rise time, and call fall time (Appendixes S2 and S3). We also counted the number of pulses inside a call as the pulse number. For the spectral properties, we measured the largest peak on the power spectrum (FFT = 1024 points, Hanning window) as the dominant frequency, the frequencies at the 25% and 75% of the power spectrum as the 1st and 3rd quartile frequencies, and the difference in frequency between the 1st and 3rd quartiles as the IQR bandwidth of frequencies (Appendixes S2 and S3). Individual call parameters were obtained by averaging the 20 calls from the same caller.

### DNA sequencing and analyses

DNA was extracted with the EasyPure^™^ Gel/PCR DNA Fragments Extraction Kit (Bioman Scientific Co., Ltd, Taipei, Taiwan). The *COI* sequence was amplified using primer LCO020 (5′ TTT CAA CCA ACC ACA AAG ACA TGG G 3′) and HCO710 (5′ TAT ACT TCA GGG TGG CCA AAR AAT CA 3′) via polymerase chain reactions (PCR). We performed double‐stranded PCR in 20 *μ*l volume with the following steps: 1 cycle denaturation at 94°C for 3 min, followed by 35 cycles at 94°C for 30 s, annealing at 50°C for 40 s, and 72°C for 60 s, with a final elongation at 72°C for 10 min in an iCycler Thermal Cycler (Bio‐Rad, Richmond, CA, USA). The PCR products were run on 1.2% agarose gels in 1×TBE buffer to confirm that the lengths of the particular fragments were correctly amplified. Automated PCR products were carried out with the same primers in both directions on ABI 3730 by Genomics BioSci & Tech Corp. (Taipei, Taiwan).

The sequences were edited by Sequencher v4.9 (GeneCode, Boston, MA) and aligned by ClustalX (Jeanmougin et al. 1998), which produced 674 base pairs. We used MEGA v6.0 (Tamura et al. 2013) to estimate the best‐fit model (Kimura‐2‐parameter in this case) for the phylogenetic relationship, and we calculated the pairwise genetic distances between populations (Appendix S4). We calculated the genetic differentiation (*F*_st_) among the populations using DnaSP v5.1 (Librado and Rozas 2009). We constructed haplotype neighbor‐joining network using Network v4.6 (Bandelt et al. 1999).

### Statistical analyses

We used the principal component analysis to condense the 10 call properties into two independent variables that together explained 67% of total variance. The first principal component (PC1) reflects the temporal properties, whereas the second (PC2) reflects the spectral properties (Table 1). We used the scaled mass index (SMI) to represent body size, which was calculated from SVLs and body weight according to the equation: SMI $\left({\hat{M}}_{l}\right)=M_{i}{\left[\frac{L_{0}}{L_{i}}\right]}^{b_{\mathrm{SMA}}}$, where *M*_*i*_ and *L*_*i*_ are the body mass and the linear body measurement of individual *i*, respectively; *b*_SMA_ is the scaling exponent estimated by the SMA regression of *M* on *L*;* L*_0_ is an arbitrary value of *L* (the arithmetic mean value for the study populations); and ${\hat{M}}_{l}$ is the predicted body mass for individual *i* when the linear body measure is standardized to *L*_0_ (Peig and Green 2009, 2010). We examined geographic variation in the two PCs using a general linear mixed model, with sample locality as the fixed effect, and ambient temperature at the time of call recording and the caller's SMI as two covariates. We examined geographic variation in SMI scores using a general linear model, with sample locality as the fixed effect. The relationships between the mean PCs of the populations and long‐term mean temperatures at the localities, as well as that between the mean PCs and mean SMI scores of the populations, were tested using the Pearson correlation.

**Table 1:** The loadings of the first two principal components of the advertisement calls of *Microhyla fissipes*

Call variables	Principal component	
	PC1	PC2
Duration (s)	0.4821	0.0324
Rise time (s)	0.4435	0.1359
Fall time (s)	0.4146	−0.1459
Interval (s)	0.3661	0.0938
Call rate	−0.4579	−0.0480
Pulse number	0.2156	−0.0503
Dominant frequency (Hz)	−0.0318	0.5545
1st quartile frequency (Hz)	−0.0267	0.5729
3rd quartile frequency (Hz)	−0.0739	0.5041
IQR bandwidth of frequency (Hz)	−0.0396	−0.2362

To test RCD, we compared the differences in the PCs between allopatric and sympatric populations of *M. fissipes* using a general linear mixed model. We included the type of populations (sympatry or allopatry) as the fixed effect, ambient temperatures and SMI scores as two covariates, and sample locality nested in the type of populations as a random effect. To test genetic drift, we extracted the residuals from the linear regressions of the PCs against ambient temperatures and SMI scores to obtain the standardized call properties (PC1 = 13.51−0.58 × temperature + 0.025 × SMI; PC2 = 1.71−0.056 × temperature − 0.30 × SMI). We then used the residual PCs to calculate the dissimilarity matrices of the pairwise differences in the call properties among *M. fissipes* populations. We also calculated the shortest pairwise geographic distances between the sample localities using their geographic coordinates. Finally, we conducted Mantel tests with 1000 permutations to test correlations between acoustic differences, geographic distances, and genetic distances. We applied phenotypic *Q*_st_(*P*_st_) − *F*_st_ comparison (Spitze 1993) to distinguish the effect of selection from genetic drift on acoustic variation (Storz 2002; Sæther et al. 2007). When *P*_st_ of acoustic traits (PC1 and PC2) equals genetic *F*_st_, the pattern of acoustic variation is likely explained by genetic drift. If *P*_st_ is greater than *F*_st_, directional selection or disruptive selection might contribute to the acoustic variation. When *P*_st_ is smaller than *F*_st_, the pattern suggests the effect of uniform selection or stabilizing selection (Storz 2002; Sæther et al. 2007). We estimated *P*_st_ with phenotypic data using the equation: $P_{\mathrm{st}}=\sigma_{b}^{2}/\sigma_{b}^{2}+2\sigma_{w}^{2}$, where $\sigma_{b}^{2}$ represents the observed variance among populations and $\sigma_{w}^{2}$ represents the observed variance within population (Appendix S5). Matrix permutation test using the Mantel–Haenszel method with 1000 permutations was conducted to compare the quantitative traits (*P*_st_), the two acoustic PCs, to the neutral marker (*F*_st_). Because we did not have SMI scores for three individuals, all statistical analyses involving individual SMI scores would have a total sample of 143 instead of 146. All statistical tests were conducted using either SAS 9.3 (SAS Institute Inc., Cary, NC) or R 3.11 (R Foundation for Statistical Computing, Vienna, Austria).

## Results

### Phylogenetic relationship among geographic populations of *M. fissipes*

A total of 24 haplotypes were obtained from *M. fissipes*, with a haplotype diversity (*h*) of 0.88. The *M. fissipes* populations are classified into four groups (Fig. 2B): the northern clade (2 populations), the western clade (4 populations), the eastern clade (4 populations; note that this group is not strictly monophyletic), and the southern clade (1 population; note that this population is mixed with a few individuals carrying the western and eastern haplotypes). The result of minimum spanning network suggests a congruent pattern of the phylogenetic tree, with the four major lineages separated by several mutation steps (Fig. 2C).

### Geographic variation in the advertisement calls and body size

**Figure 3:**
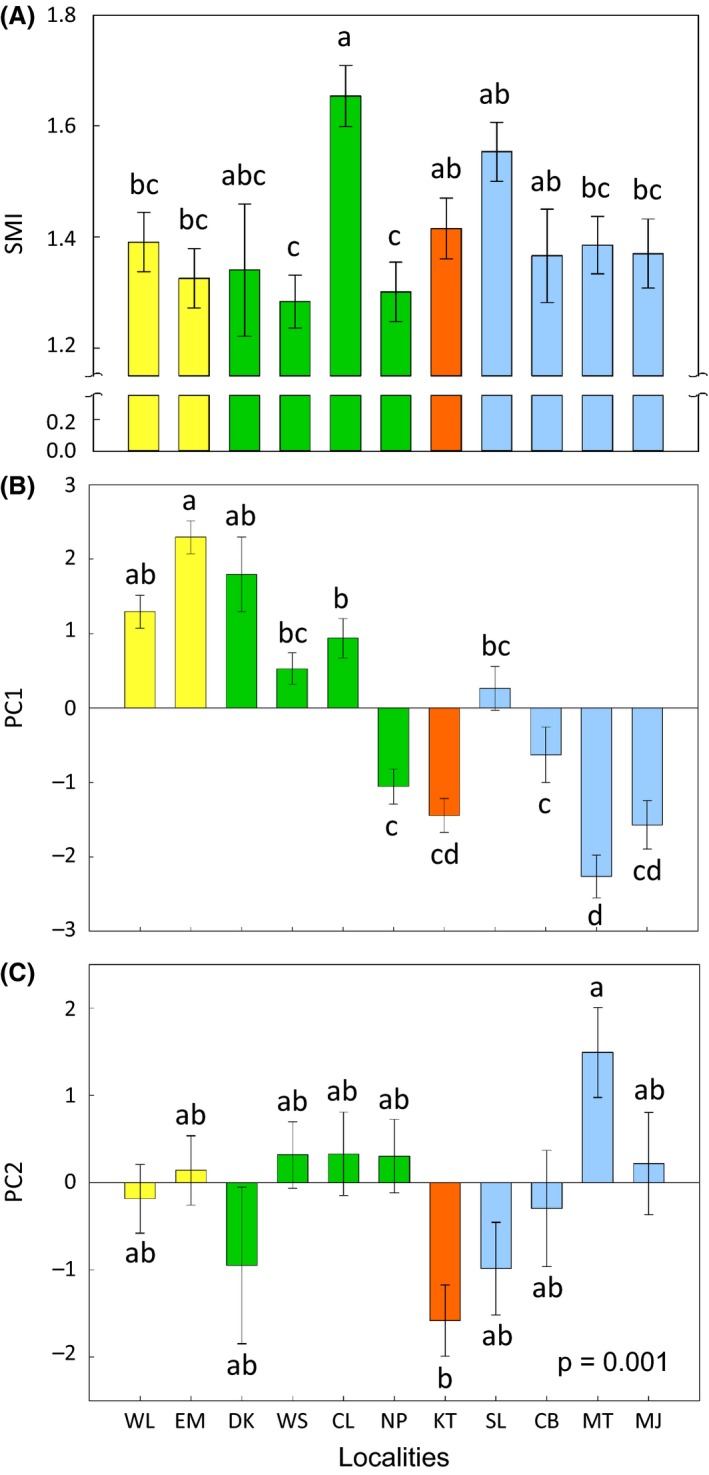
Body size, temporal, and spectral properties of the advertisement calls of *Microhyla fissipes*. (A) Body size represented by the scaled body mass index (SMI). (B) Temporal properties of the calls represented by PC1. (C) Spectral properties of the calls represented by PC2. The colors of the bars correspond to the four phylogenetic clades (Fig. 2B and C). Different letters denote significant differences in call properties among populations (post hoc comparisons with Tukey–Kramer adjustment). The error bars denote one standard error of the least‐square means.

Both the temporal (PC1) and spectral (PC2) properties of the advertisement calls of *M. fissipes* varied among geographic populations (Table 2; Fig. 3B and C). The body size (SMI) also varied among *M. fissipes* populations (*F*_10,132_ = 4.19, *P *<* *0.0001; Fig. 3A). The geographic variation of the temporal properties of the calls reflected the phylogenetic structure of *M. fissipes* to some degree, with the northern and eastern clades showing the most divergent calls (Appendix S6). The spectral properties of the calls and SMI scores, however, did not reflect the phylogenetic structure (Appendix S6).

**Table 2:** Geographic variation in the advertisement calls of *Microhyla fissipes*

Effect	Num DF	Den DF	PC1		PC2	
			*F*	*P*	*F*	*P*
Sample locality	10	130	29.73	<0.0001	3.17	0.001
Ambient temperature	1	130	19.01	<0.0001	4.05	0.05
SMI	1	130	0.61	0.44	0.09	0.77

PC1 is the temporal properties and PC2 the spectral properties of the calls. Ambient temperature is the temperature at the time of call recording, and SMI is the scaled body mass index of the caller.

### Ecological correlates of advertisement calls

Neither the temporal (PC1) nor spectral (PC2) properties of the advertisement calls of *M. fissipes* correlated with long‐term average temperature at the localities (PC1: *r *=* *0.15, *P *=* *0.66; PC2: *r *=* *−0.18, *P *=* *0.60; *n* = 11). Similarly, neither of the two call properties correlated with mean SMI scores of the populations (PC1: *r *=* *0.19, *P *=* *0.58; PC2: *r *=* *0.02, *P *=* *0.95; *n* = 11).

### Reproductive character displacement in advertisement calls

Neither the temporal properties (PC1) nor the spectral properties (PC2) of the advertisement calls differed between the sympatric and allopatric populations (Table 3), indicating a lack of RCD in the advertisement calls of *M. fissipes*.

**Table 3:** Differences in the advertisement calls of *Microhyla fissipes* between populations sympatric with and allopatric to *M. heymoni*

Effect	Num DF	Den DF	PC1		PC2	
			*F*	*P*	*F*	*P*
Sympatry versus allopatry	1	9	0.28	0.61	0.65	0.44
Ambient temperature	1	130	23.75	<0.0001	1.50	0.22
SMI	1	130	0.66	0.42	0.14	0.70

PC1 is the temporal properties and PC2 the spectral properties of the calls. Ambient temperature is the temperature at the time of call recording, and SMI is the scaled body mass index of the caller. The sample locality nested in the type of population (sympatry vs. allopatry) was included as a random effect.

### Geographic distance, genetic distance and call divergence

**Figure 4:**
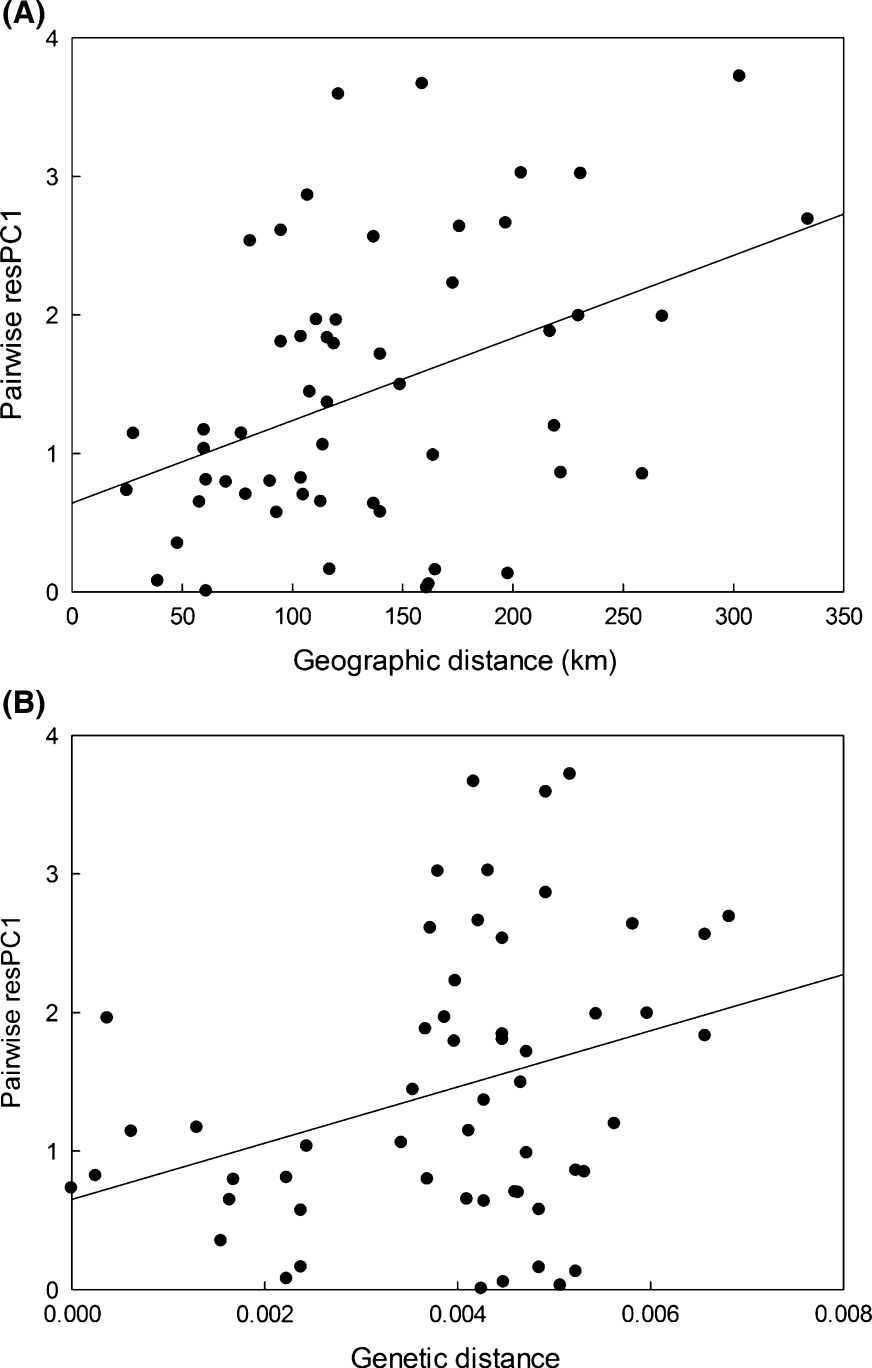
Correlations between acoustic divergence, geographic distance, and genetic distance among *Microhyla fissipes* populations. (A) Correlation between acoustic difference in temporal properties of the calls (residual PC1, standardized by temperature and SMI) and geographic distance. (B) Correlation between acoustic difference in temporal properties of the calls (residual PC1, standardized by temperature and SMI) and genetic distance.

The genetic and geographic distances among *M. fissipes* populations were positively correlated (*r = *0.64, *P *=* *0.001, *n* = 146), indicating the presence of isolation by distance effect. Furthermore, the pairwise differences in the temporal properties of the advertisement calls (residual PC1) and geographic or genetic distances were also positively correlated (*r*_*resPC1 vs. geographic distance*_ = 0.41, *P *=* *0.013; *r*_*resPC1 vs. genetic distance*_ = 0.32, *P *=* *0.028; *n* = 146; Fig. 4), suggesting that the temporal properties of the advertisement calls are likely under the influence of genetic drift. The correlations between pairwise differences in the spectral properties of the calls (PC2) and geographic or genetic distances, on the other hand, were not significant (*r*_*resPC2 vs. geographic distance*_ = 0.080, *P *=* *0.31; *r*_*resPC2 vs. genetic distance*_ = 0.083, *P *=* *0.31; *n* = 146). The matrix permutation test did not show significant differences between the quantitative traits (*P*_st_, Appendix S5) and the neutral marker (*F*_st_, Appendix S4) (*Z*_*PstPC1 vs. Fst*_ = 26.07, *P *=* *0.25; *Z*_*PstPC2 vs. Fst*_ = 12.65, *P *=* *0.19). Both the relationship test and *P*_st_‐*F*_st_ comparisons suggest that the acoustic variation of *M. fissipes* was likely caused by genetic drift.

## Discussion

This study provides an empirical case for which geographic variation of a sexual trait (i.e., advertisement calls) in a frog is likely driven by a random process (genetic drift) rather than selection (i.e., local adaptation to temperature, constraint of body size, reproductive character displacement). For explosive‐breeding or lek‐breeding frogs, it has been suggested that male quality may be related to chorus attendance, and females that mate randomly are likely to mate with high‐quality males and thereby gain fitness without incurring costs of mate assessment (Friedl and Klump 2005). Under this hypothesis, females may not exert strong selection on male calls. Although we did not test female preference for local versus foreign calls, we did show that male call properties were not correlated with body size in *M. fissipes*, suggesting that male call properties may not reflect male quality. In conclusion, we suggest that the geographic variation in the advertisement calls of *M. fissipes* is mainly shaped by drift, not ecology. Given their widespread distributions in lowland habitats throughout the subtropical and tropical Asia, *M. fissipes* and *M. heymonsi* can serve as valuable model species for studying contemporary evolution of acoustic signals under rapid environmental changes.

### Reproductive character displacement did not occur in *M. fissipes*

*Microhyla fissipes* did not exhibit different advertisement calls between the allopatric and sympatric populations, indicating a lack of reproductive character displacement in *M. fissipes* in response to the presence of *M. heymonsi*. Nevertheless, reproductive isolation between these two *Microhyla* species is apparently effective. No hybrids of these two species have ever been reported in the wild (Lin 2009; this study). Although *M. fissipes* tend to produce shorter advertisement calls with a higher call rate and a higher pulse number than *M. heymonsi* (Appendix S3), whether this amount of difference is sufficient for the females to distinguish between them in a mixed‐species chorus remains unclear. Alternatively, mate recognition in these two species may have been achieved through visual cues. Anurans as a group are highly sensitive to visual signals that are used for predation avoidance, communication, and mate choice, even in a low luminous environment (Aho et al. 1988, 1993; Cummings et al. 2008; Preininger et al. 2013). In some frog species, visual cues have been demonstrated to be as important as acoustic cues for female choice (Candolin 2003; Gomez et al. 2009). *Microhyla fissipes* has a distinct dark brown patch on their dorsal side. By contrast, *M. heymonsi* is generally light brown to gray on their dorsal side, with a thin yellow line in the middle. Therefore, visual cues might be useful to distinguish the mates at a close distance. Advertisement calls, on the other hand, might be used as an aggregation signal for the females to locate the breeding assemblage (Gerhardt and Huber 2002).

### Drift explains acoustic divergence in *M. fissipes*

Genetic drift has been reported as one of the major forces shaping acoustic variation in several amphibian cases such as *Phylloscopus trochiloides*,* Dendropsophus ebraccatus*,* Scotinomys teguina*, or *Hyla japonica* (Irwin et al. 2008; Ohmer et al. 2009; Campbell et al. 2010; Jang et al. 2011). For *M. fissipes*, we also found support for a role of genetic drift in their acoustic divergence. It is often difficult to differentiate between genetic and culture drift. Previous studies suggest that cultural drift, the phenomenon caused by learning from adjacent members, may explain widespread acoustic divergence at interspecific or population levels (Tyack 2008; Sewall 2009; Sun et al. 2013; Lin et al. 2014). However, cultural drift should lead to correlations between acoustic difference and geographic distance, but not necessarily correlations between acoustic difference and genetic distance. Therefore, cultural drift alone may not be sufficient to explain the observed acoustic divergence in *M. fissipes*.

Although we provided a likely case for neutral acoustic differentiation of *M fissipes* in this study, we cautioned that not all possible ecological drivers were exhausted. In some cases, acoustically orienting predator (Ryan et al. 1982; Akre et al. 2011), habitat structure (Ryan and Wilczynski 1991), or vocal anatomy (Fitch and Hauser 2003; Gridi‐Papp et al. 2006) have been suspected to have contributed to acoustic differentiation.

### Can acoustic divergence facilitate diversification in frogs of Taiwan?

Acoustic divergence in mating calls among populations could reduce gene flow and facilitate population differentiation and speciation. The island of Taiwan is known for its steep topography and a highly fragmented landscape, which provide the backdrop for rapid genetic differentiation across very short distances (Lin et al. 2012; Tseng et al. 2015). Herptile taxa, with their limited dispersal ability, might be particularly sensitive to such landscapes. In recent years, several cryptic herptile species have been reported in Taiwan (Lue and Lin 2008; You et al. 2015), and two tree frogs have been shown to exhibit extremely high within‐island divergence (*Buergeria robusta*, Lin et al. 2012; *B. japonica*, Tominaga et al. 2015). Although the genetic divergence among *M. fissipes* populations is not as high as the *Buergeria* frogs, our data suggest that the temporal properties of *M. fissipes* advertisement calls diverged between the northern and eastern phylogenetic clades (Appendix S6), which could potentially contribute to their contemporary speciation.

Speciation through acoustic divergence in mating calls relies on effective assortative mating between populations (Smith 1966; Hoskin et al. 2005). Therefore, further research that could confirm the presence of assortative mating in *M. fissipes* among populations with divergent advertisement calls is urgently needed.

## Data Accessibility

The data on the original 10 call properties of the 146 *Microhyla fissipes* and 87 *M. heymonsi*, as well as the body weights and snout‐vent lengths of the 143 *M. fissipes*, can be found on Dryad (DOI: doi:10.5061/dryad.g8d5k); the mitochondrial *COI* sequences can be found on GenBank (KT894589‐KT894670).

## Conflict of Interest

None declared.

## Supporting information


**Appendix S1.** Locality, abbreviation, latitude, longitude, elevation, mean temperature and sample sizes of *Microhyla fissipes* and *M. heymonsi* for each sample locality used in this study.
**Appendix S2.** Illustrations of the temporal and spectral properties of the advertisement call of a *Microhyla fissipes* individual.
**Appendix S3.** Acoustic characteristics of the advertisement calls of *Microhyla fissipes* and *M. heymonsi*.
**Appendix S4.** Pairwise comparisons of genetic differentiation (*F*_st_) and genetic distance (Kimura‐2‐parameter) among populations of *Microhyla fissipes*.
**Appendix S5.** Pairwise comparisons of *P*_st_ of first principal component (PC1) and second principal component (PC2) of acoustic characters among *Microhyla fissipes* populations.
**Appendix S6.** Geographic variation in the advertisement calls and body size of *Microhyla fissipes* among different regional clades in Taiwan.
