# Supplementary material for: Geographic variation in advertisement calls of a Microhylid frog – testing the role of drift and ecology
Source: Ecol Evol. 2016 Apr 12;6(10):3289–98. doi: 10.1002/ece3.2116 (PMC4833500; doi:10.1002/ece3.2116)
Supplement: Supplementary file 1 — Appendix S1. Locality, abbreviation, latitude, longitude, elevation, mean temperature and sample sizes of Microhyla fissipes and M. heymonsi for each sample locality used in this study. Appendix S2. Illustrations of the temporal and spectral properties of the advertisement call of a Microhyla fissipes individual. Appendix S3. Acoustic characteristics of the advertisement calls of Microhyla fissipes and M. heymonsi. Appendix S4. Pairwise comparisons of genetic differentiation (F st) and genetic distance (Kimura‐2‐parameter) among populations of Microhyla fissipes. Appendix S5. Pairwise comparisons of P st of first principal component (PC1) and second principal component (PC2) of acoustic characters among Microhyla fissipes populations. Appendix S6. Geographic variation in the advertisement calls and body size of Microhyla fissipes among different regional clades in Taiwan. [file ECE3-6-3289-s001.docx]

**Supporting Information**

**Geographic variation in advertisement calls of a Microhylid frog—Testing the role of drift and ecology**

Ko-Huan Lee^1^, Pei-Jen L. Shaner^1^, Yen-Po Lin^2^ & Si-Min Lin^1*^

^1^ Department of Life Science, National Taiwan Normal University, Taipei, Taiwan

^2^ Division of Zoology, Taiwan Endemic Species Research Institute, Nantou, Taiwan

* Corresponding Author

Email: [fish@ntnu.edu.tw](mailto:fish@ntnu.edu.tw); [fishdna@ms31.hinet.net](mailto:fishdna@ms31.hinet.net)

**Appendix S1.** Locality, abbreviation, latitude, longitude, elevation, mean temperature and sample sizes of *Microhyla fissipes* and *M. heymonsi* for each sample locality used in this study.

| Locality | Abbreviation | Latitude | Longitude | Elevation | Mean temp.* | Sample Size | |
| --- | --- | --- | --- | --- | --- | --- | --- |
|  |  |  |  |  |  | *M. fissipes* | *M. heymonsi* |
| Wulai | WL | 24.8898 | 121.5672 | 395 m | 23.5 °C | 15 | 0 |
| Ermei | EM | 24.6872 | 121.0135 | 85 m | 24.0 °C | 15 | 0 |
| Dakeng | DK | 24.1726 | 120.7852 | 476 m | 25.6 °C | 3 | 8 |
| Wushanto | WS | 23.2383 | 120.3753 | 46 m | 24.2 °C | 19 | 9 |
| Chungliao | CL | 22.8083 | 120.4041 | 123 m | 25.6 °C | 17 | 18 |
| Neipu | NP | 22.6393 | 120.6025 | 100 m | 24.5 °C | 15 | 18 |
| Kenting | KT | 21.9548 | 120.8197 | 191 m | 26.1 °C | 14 | 0 |
| Shanli | SL | 22.8525 | 121.1504 | 168 m | 23.8 °C | 15 | 0 |
| Changbin | CB | 23.3053 | 121.4236 | 170 m | 23.3 °C | 6 | 15 |
| Mataian | MT | 23.6589 | 121.4130 | 110 m | 24.8 °C | 16 | 0 |
| Mijian | MJ | 23.8470 | 121.5421 | 98 m | 22.2 °C | 11 | 0 |
| Huben | HB | 23.7266 | 120.6212 | 222 m | 24.1 °C | 0 | 9 |
| Fuyuan | FY | 23.5889 | 121.4012 | 191 m | 24.7 °C | 0 | 7 |

*Mean temperature is the monthly average temperature between March and August from 1960 to 2009 (extracted from Taiwan Climate Change Projection and Information Platform, <http://tccip.ncdr.nat.gov.tw>).

**Appendix S2.** Illustrations of the temporal and spectral properties of the advertisement call of a *Microhyla fissipes* individual. (A) 15 consecutive calls from one individual, where the number of calls within a period is defined as the call rate; (B) each call is composed of a series of pulses, where the number of pulses in a call is defined as the pulse number; (C) the amplification of a pulse; (D) the power spectrum of a single call.

**Appendix S3.** Acoustic characteristics of the advertisement calls of *Microhyla fissipes* and *M. heymonsi*.

| Acoustic traits | *Microhyla fissipes* (N = 146) | |  | *Microhyla heymonsi* (N = 87) | |  |
| --- | --- | --- | --- | --- | --- | --- |
|  | Mean | Range |  | Mean | Range |  |
| Call duration (s) | 0.284 ± 0.005 | 0.178–0.401 |  | 0.384 ± 0.006 | 0.250–0.602 |  |
| Call interval (s) | 0.401 ± 0.020 | 0.196–0.721 |  | 0.860 ± 0.036 | 0.390–2.761 |  |
| Call rate (N/m) | 92.940 ± 1.415 | 55.875–143.743 |  | 54.292 ± 1.833 | 29.843–104.857 |  |
| Call rise time (s) | 0.164 ± 0.003 | 0.089–0.236 |  | 0.182 ± 0.004 | 0.118–0.308 |  |
| Call fall time (s) | 0.119 ± 0.002 | 0.079–0.169 |  | 0.202 ± 0.003 | 0.128–0.295 |  |
| Pulse number (N) | 13.8 ± 0.1 | 10.0–17.0 |  | 11.7 ± 0.1 | 10.0–15.0 |  |
| Dominant frequency (Hz) | 2784.85 ± 24.10 | 1119.7–3445.30 |  | 2902.27 ± 31.22 | 1356.60–3359.20 |  |
| 1^st^ quartile frequency (Hz) | 2431.05 ± 24.16 | 1248.90–3316.10 |  | 2609.23 ± 31.30 | 1550.40–2950.05 |  |
| 3^rd^ quartile frequency (Hz) | 2869.66 ± 17.45 | 1894.90–3789.80 |  | 2986.41 ± 22.61 | 2584.00–3359.20 |  |
| IQR bandwidth frequency (Hz) | 439.22 ± 19.76 | 129.20–1335.10 |  | 378.69 ± 25.60 | 129.20–1378.10 |  |

**Appendix S4.** Pairwise comparisons of genetic differentiation (F_st_, upper-right) and genetic distance (Kimura-2-parameter, lower-left) among populations of *Microhyla fissipes*. Locality names are abbreviated (for the full names and geographic coordinates of the localities, see Table S1).

|  | WL | EM | DK | WS | CL | NP | KT | SL | CB | MT | MJ |
| --- | --- | --- | --- | --- | --- | --- | --- | --- | --- | --- | --- |
| WL |  | 0.167 | 0.686 | 0.514 | 0.654 | 0.639 | 0.409 | 0.608 | 0.598 | 0.758 | 0.758 |
| EM | 0.00244 |  | 0.870 | 0.601 | 0.811 | 0.784 | 0.442 | 0.724 | 0.714 | 0.909 | 0.909 |
| DK | 0.00507 | 0.00342 |  | 0.122 | 0.000 | 0.000 | 0.424 | 0.808 | 0.800 | 0.999 | 0.999 |
| WS | 0.00563 | 0.00398 | 0.00130 |  | 0.103 | 0.095 | 0.232 | 0.488 | 0.466 | 0.733 | 0.733 |
| CL | 0.00532 | 0.00367 | 0.00025 | 0.00155 |  | 0.000 | 0.400 | 0.759 | 0.750 | 0.947 | 0.947 |
| NP | 0.00544 | 0.00380 | 0.00037 | 0.00168 | 0.00062 |  | 0.389 | 0.737 | 0.727 | 0.923 | 0.923 |
| KT | 0.00682 | 0.00517 | 0.00422 | 0.00466 | 0.00447 | 0.00460 |  | 0.312 | 0.289 | 0.533 | 0.533 |
| SL | 0.00597 | 0.00432 | 0.00387 | 0.00369 | 0.00412 | 0.00425 | 0.00164 |  | 0.091 | 0.688 | 0.688 |
| CB | 0.00582 | 0.00417 | 0.00372 | 0.00354 | 0.00397 | 0.00410 | 0.00238 | 0.00463 |  | 0.667 | 0.667 |
| MT | 0.00657 | 0.00492 | 0.00447 | 0.00428 | 0.00472 | 0.00485 | 0.00238 | 0.00448 | 0.00223 |  | 0.000 |
| MJ | 0.00657 | 0.00492 | 0.00447 | 0.00428 | 0.00472 | 0.00485 | 0.00164 | 0.00523 | 0.00223 | 0.00000 |  |

**Appendix S5.** Pairwise comparisons of P_st_ of first principal component (PC1, upper-right) and second principal component (PC2) of acoustic characters among *Microhyla fissipes* populations. See the content for definition and calculation of P_st_. Locality names are abbreviated as listed in Table A1.

|  | WL | EM | DK | WS | CL | NP | KT | SL | CB | MT | MJ |
| --- | --- | --- | --- | --- | --- | --- | --- | --- | --- | --- | --- |
| WL |  | 0.833 | 0.726 | 0.464 | 0.370 | 0.983 | 0.968 | 0.141 | 0.894 | 0.994 | 0.991 |
| EM | 0.293 |  | 0.001 | 0.868 | 0.607 | 0.986 | 0.977 | 0.855 | 0.932 | 0.994 | 0.990 |
| DK | 0.236 | 0.422 |  | 0.640 | 0.322 | 0.972 | 0.929 | 0.717 | 0.916 | 0.988 | 0.985 |
| WS | 0.457 | 0.239 | 0.288 |  | 0.687 | 0.951 | 0.936 | 0.267 | 0.575 | 0.985 | 0.971 |
| CL | 0.807 | 0.565 | 0.812 | 0.001 |  | 0.979 | 0.968 | 0.526 | 0.872 | 0.992 | 0.986 |
| NP | 0.224 | 0.019 | 0.432 | 0.293 | 0.662 |  | 0.020 | 0.978 | 0.884 | 0.959 | 0.881 |
| KT | 0.620 | 0.701 | 0.149 | 0.753 | 0.819 | 0.689 |  | 0.961 | 0.783 | 0.916 | 0.746 |
| SL | 0.108 | 0.509 | 0.170 | 0.546 | 0.880 | 0.467 | 0.579 |  | 0.837 | 0.992 | 0.987 |
| CB | 0.304 | 0.015 | 0.539 | 0.087 | 0.403 | 0.057 | 0.517 | 0.525 |  | 0.977 | 0.960 |
| MT | 0.757 | 0.485 | 0.622 | 0.041 | 0.147 | 0.639 | 0.816 | 0.819 | 0.358 |  | 0.757 |
| MJ | 0.143 | 0.377 | 0.003 | 0.489 | 0.712 | 0.340 | 0.376 | 0.054 | 0.270 | 0.698 |  |


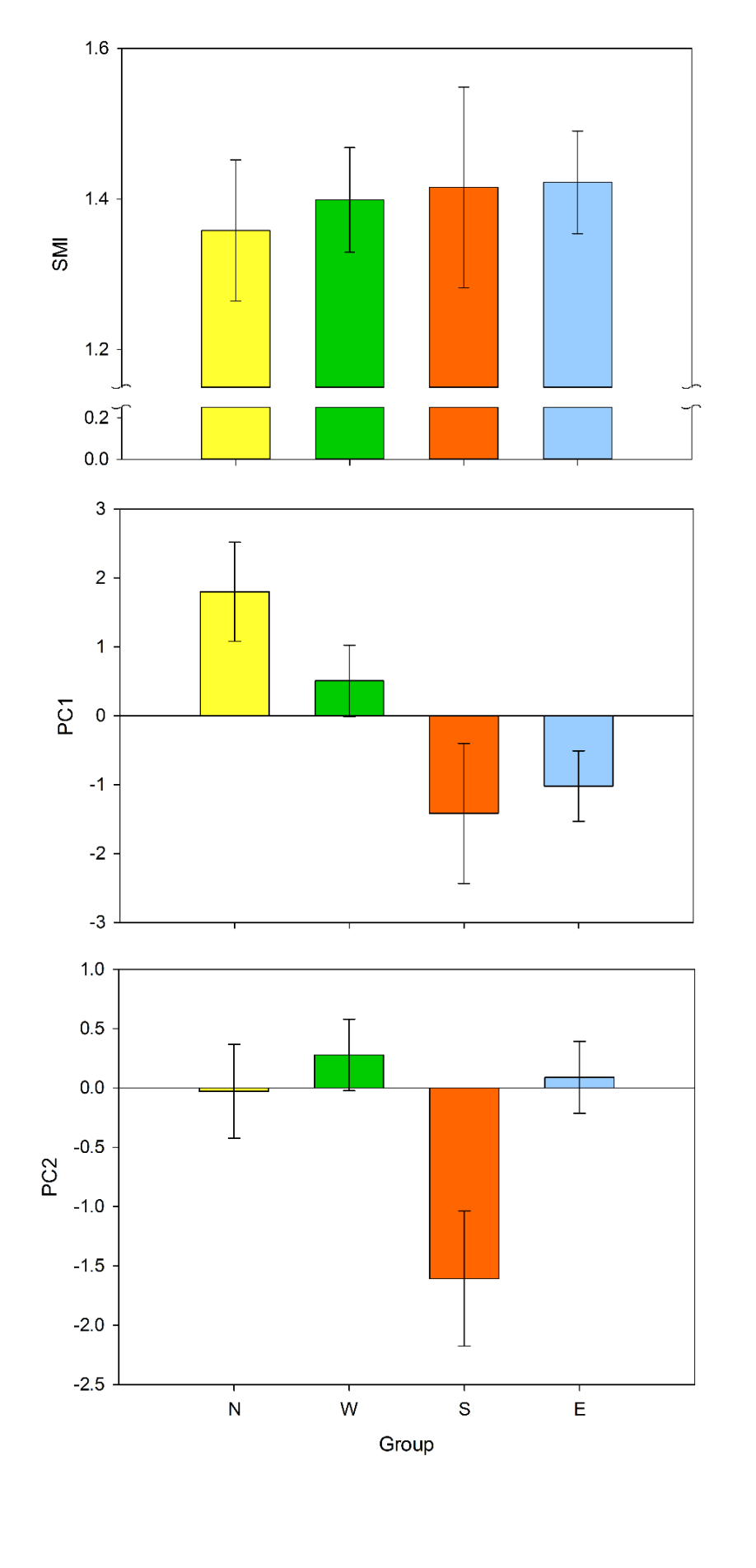


**Appendix S6.** Geographic variation in the advertisement calls and body size of *Microhyla fissipes* among different regional clades in Taiwan. The four phylogenetic clades of *M. fissipes* are: the northern clade (2 populations), the western clade (4 populations), the eastern clade (4 populations), and the southern clade (1 population). The advertisement calls include the temporal properties (principal component 1) and spectral properties (principal component 2). The body size is represented by the scaled mass index (SMI), which is a function of the snout-vent lengths and body weight. See *Material and methods* of the main text for details.
